# Rosemary (*Rosmarinus officinalis*) Extract Modulates CHOP/GADD153 to Promote Androgen Receptor Degradation and Decreases Xenograft Tumor Growth

**DOI:** 10.1371/journal.pone.0089772

**Published:** 2014-03-05

**Authors:** Sakina M. Petiwala, Saba Berhe, Gongbo Li, Angela G. Puthenveetil, Ozair Rahman, Larisa Nonn, Jeremy J. Johnson

**Affiliations:** 1 Department of Pharmacy Practice, College of Pharmacy, University of Illinois at Chicago, Chicago, Illinois, United States of America; 2 University of Illinois Cancer Center, University of Illinois at Chicago, Chicago, Illinois, United States of America; 3 Department of Pathology, College of Medicine, University of Illinois at Chicago, Chicago, Illinois, United States of America; Roswell Park Cancer Institute, United States of America

## Abstract

The Mediterranean diet has long been attributed to preventing or delaying the onset of cardiovascular disease, diabetes and various solid organ cancers. In this particular study, a rosemary extract standardized to carnosic acid was evaluated for its potential in disrupting the endoplasmic reticulum machinery to decrease the viability of prostate cancer cells and promote degradation of the androgen receptor. Two human prostate cancer cell lines, 22Rv1 and LNCaP, and prostate epithelial cells procured from two different patients undergoing radical prostatectomy were treated with standardized rosemary extract and evaluated by flow cytometry, MTT, BrdU, Western blot and fluorescent microscopy. A significant modulation of endoplasmic reticulum stress proteins was observed in cancer cells while normal prostate epithelial cells did not undergo endoplasmic reticulum stress. This biphasic response suggests that standardized rosemary extract may preferentially target cancer cells as opposed to “normal” cells. Furthermore, we observed standardized rosemary extract to decrease androgen receptor expression that appears to be regulated by the expression of CHOP/GADD153. Using a xenograft tumor model we observed standardized rosemary extract when given orally to significantly suppress tumor growth by 46% compared to mice not receiving standardized rosemary extract. In the last several years regulatory governing bodies (e.g. European Union) have approved standardized rosemary extracts as food preservatives. These results are especially significant as it is becoming more likely that individuals will be receiving standardized rosemary extracts that are a part of a natural preservative system in various food preparations. Taken a step further, it is possible that the potential benefits that are often associated with a “Mediterranean Diet” in the future may begin to extend beyond the Mediterranean diet as more of the population is consuming standardized rosemary extracts.

## Introduction

The androgen receptor (AR) has been a target for prostate cancer for a longtime, however, there are limitations as to why anti-androgens are only effective for a short period of time. First, there are a variety of clinical adverse events (e.g. osteoporosis, hypercholesteremia, hyperglycemia) and second, the accumulating mutation profile within the androgen receptor rendering them ineffective [1], [2], [3]. About 85% of prostate cancer patients will have a favorable response with anti-androgen pharmacotherapy, however, the benefits of anti-androgens are short. Very quickly, molecular modifications occur within the AR resulting in anti-androgen resistance in more than 50% of the cases [4], [5]. There has been significant progress in the development of anti-androgens that are effective in castration resistant prostate cancers including enzalutamide (formerly known as MDV3100) [6]. The future of this agent and other similar analogues appears to be promising, however, it is important that additional therapeutic strategies be developed to combat prostate cancer.

The increased proliferation of prostate cancer cells as well as other cancers is highly dependent on increased activity of the endoplasmic reticulum to successfully fold, assemble, and transport proteins [7], [8], [9]. This process is so critical that if not regulated properly cells will undergo apoptosis. As the demand for cellular protein increases, thereby altering ER homeostasis, there is also an increase in “translational sloppiness” generating proteins that are unfolded or misfolded. Left unchecked, these cells would typically undergo apoptosis, however, there is a concerted effort to prevent apoptosis and “re-establish” ER homeostasis. To accomplish this cancer cells utilize a signal transduction pathway known as the “unfolded protein response (UPR)”. Traditionally, the unfolded protein response directed by proteins that include CHOP, IRE1α, PERK as well as others is to promote cell survival [7]. There are clearly instances where this is true, however, there is evidence linking proteins associated with the UPR in promoting degradation of key proteins [10], [11].

The unfolded protein response is closely associated with degradation of key proteins and represents an important therapeutic strategy for prostate cancer. The targeted degradation of the androgen receptor represents an opportunity to circumvent the traditional obstacles that occur with androgen receptor antagonists including molecular modifications to promote resistance. As evidenced by our studies included herein, we present data suggesting that a significant increase in ER stress occurs following treatment with rosemary extract leading to androgen receptor degradation in prostate cancer cells. This targeted degradation of the androgen receptor appears to be critically regulated by the ER stress protein CHOP/GADD153 and ER chaperone BiP. Herein, we provide evidence that CHOP/GADD153 and BiP activation is essential for degradation of the androgen receptor in prostate cancer.

## Materials and Methods

The Bax, PERK, IRE1α, BiP, CHOP and β-actin primary antibodies were obtained from Cell Signaling Technology. The androgen receptor (AR) and caspase-4 primary antibody and the anti-mouse and anti-rabbit HRP conjugated secondary antibodies were obtained from Santa Cruz Biotechnology, Inc. Anti-mouse and anti-rabbit fluorescent labeled secondary antibodies were obtained from Invitrogen. CHOP siRNA, BiP siRNA and control siRNA were purchased from Santa Cruz Biotechnology, Inc. and the *Trans*IT-siQuest reagent from Mirus Bio LLC. The APO-DIRECT Kit was obtained from Phoenix Flow Systems, BrdU Cell Proliferation Assay Kit and PathScan Cleaved Caspase-3 Sandwich ELISA Kit from Cell Signaling Technology, DeadEnd Fluorometric TUNEL System from Promega and the PSA ELISA kit from Anogen.

### Standardization of Rosemary Extract to carnosic acid

Stock solution of carnosic acid and rosemary extract was prepared by dissolving it in DMSO. Working standard solutions ranging from 2.5 µg/ml to 6.5 µg/ml for carnosic acid were diluted with acetonitrile. Rosemary extract was diluted in acetonitrile at a final concentration of 10 µg/ml (i.e. 4 µg/ml of carnosic acid). Each standard reaction contained rosmarinic acid as an internal standard at a ratio of 1∶1 and at a final concentration of 5 µg/ml. The samples were transferred to auto sampler vials and 20 µL of the sample was injected into the LC-MS/MS system for analysis. The standard curve of carnosic acid had a R^2^ value of 0.9778.

### Instrumentation and LC/MS/MS Conditions

The LC-MS/MS system consisted of an Agilent HPLC auto sampler, Agilent quaternary pump, and an API-3200 Qtrap mass spectrometer with a turbo-ion spray source (Applied Biosystem, Foster City, CA). The Q-trap was equipped with an electrospray ionization (ESI) source and operated in the negative-ion mode. Total eluent flow from the LC was directed to the turbo ion spray source without splitting. A valco valve was used to divert the first two minutes of the eluent to waste. Needle voltage was 4.5 kV, turbo ion spray heater temperature 500°C, (GS1) nebulizer gas (nitrogen) 50 psi, and (GS2) turbo heater gas (nitrogen) 50 psi. Curtain gas (nitrogen) was set at 10, and collision gas (CAD, nitrogen) pressure in the collision cell was set at medium. The optimum values for collision energy (CE), declustering potential (DP), and cell entrance potential (CEP) were optimized individually for each compound. Tandem mass spectrometry (MS/MS) optimization was established by directly infusing 100 µg/mL of analyte in acetonitrile.

The instrument was operated in unit resolution mode with the peak width (full width at half-maximum, FWHM) set to 0.7 m/z both at Q1 and Q3. The selected reaction monitoring scheme followed transitions of the precursor to selected product ions with the following values: m/z 331–287 for carnosic acid and m/z 359/161 for rosmarinic acid. Chromatography was performed on a Nova-Pak C18, 4.6 mm×150 mm, 4.0 µm analytical column (Waters, Milford, MA, USA). The mobile phase consisting of 10% ACN containing 0.1% formic acid in water and ACN (0.1%formic acid) mixed with methanol at 1∶1 ratio was delivered at a flow rate of 0.6 mL/min (injection volume 20 µL). The gradient program was as follows: 70–95% B at 0–7 min, hold for 1 min, followed by a 95–70% B from 7–8 min, followed by a re-equilibration at 70% B from 8–15 min. Data was analyzed using the Analyst Software Version 1.4.2 (Framingham, ME, USA).

### Cell culture

22Rv1 and LNCaP human epithelial prostate cancer cells were obtained from American Type Culture Collection (ATCC, Manassas, VA). The cells were cultured in RPMI 1640 medium (Cellgro) containing 10% FBS (Atlanta Biologicals), 100 U/ml penicillin and 100 µg/ml streptomycin (Cellgro) at 37° C and 5% CO_2_. For cell treatments an oil soluble rosemary extract standardized to >40% carnosic acid was used. Cells were treated with rosemary extract at concentrations ranging from 0–70 µg/ml for 24 hrs and then harvested for different experiments as described previously [12].

### Prostate Epithelial cells

Primary prostatic epithelial cells (PrE) were established from radical prostatectomy tissue at the University of Illinois at Chicago Medical Center as described previously [13]. Fresh tissue from the peripheral zone was selected by a pathologist according to an IRB protocol approved by the University of Illinois at Chicago IRB. All samples were obtained following written informed consent from the donors. Briefly, the tissue was digested in collagenase, and plated on collagen-coated dishes in PrEGM media (Lonza, Walkersville, MD) for epithelial cell outgrowth. All cells were used at secondary passage and ∼70 % confluency (cell density). PrECs were treated with 40 µg/ml of standardized rosemary extract for 24 hr followed by downstream experiments.

### MTT assay

Cell viability was determined by 3-(4, 5-dimethylthiazol-2-yl)-2, 5-diphenyltetrazolium bromide (MTT) assay as described previously [14].

### BrdU Cell Proliferation Assay

BrdU Cell Proliferation Assay Kit was used to assess the proliferation of 22Rv1 and LNCaP cells upon treatment with rosemary extract as per the manufacturer’s instructions. Briefly, cells were seeded at a concentration of 6,000 cells/well in a 96-well plate and allowed to reach 70% confluence. Cells were treated with 0 and 50 µg/ml of rosemary extract and prepared according to the protocol for analysis.

### Cell Cycle analysis

The APO-DIRECT Kit was used for measuring cell cycle by flow cytometry. Briefly, 22Rv1 and LNCaP cells were cultured in medium containing 1% FBS for 24 hr, and then treated with DMSO or 40 µg/ml rosemary extract for 24 hr. Cell fixation and staining were performed according to manufacturer’s manual. Cells were then subjected to flow cytometry for cell cycle analysis.

### Cleaved Caspase-3 ELISA

Lysates were prepared with the same lysis buffer used for Western blotting. 10–40 micrograms of lysates were analyzed using PathScan cleaved caspase-3 sandwich ELISA following the manufacturer's instructions. Lysates were mixed with 50 µL of sample diluent at a ratio of 1∶1 and incubated in antibody-coated microwell strips. One hundred microliters of cleaved caspase-3 detection antibodies were added to each well. Binding was detected with 100 µL of horseradish peroxidase–linked streptavidin antibody and 100 µL of 3,3′,5,5′-tetramethylbenzidine substrate solution. The colored reaction product was measured in a standard ELISA plate reader at 450 nm.

### Apoptosis by TUNEL assay

22Rv1 and LNCaP cells were cultured in chamber slides at a concentration of 0.1×10^6^ cells/chamber and allowed to grow to 70% confluence. Cells were then treated with 0 and 50 µg/ml rosemary extract for 24hrs. Apoptosis was detected by TUNEL assay using the DeadEnd Fluorometric TUNEL System according to the manufacturer’s instructions followed by counterstaining with DAPI.

### Human PSA ELISA

The human PSA ELISA kit was used for the quantitative determination of PSA levels in cell culture media per the manufacturer's protocol. Following collection of media, the samples were stored at −20°C until assayed for secreted PSA. A standard curve was plotted using available PSA standards (0, 2, 10, 20, 40, and 80 ng/mL) to quantify individual samples. The equation of the standard PSA plot was y = 0.0383x + 0.0718 with a R^2^ value of 0.9953.

### Immunoblot analysis

Control as well as rosemary extract treated cells were collected and whole cell lysates prepared. Protein concentrations in supernatants were measured using a BCA protein assay kit (Thermo Scientific). 10–40 µg protein was separated by SDS gel electrophoresis and transferred on nitrocellulose membrane (Millipore). The blots were blocked with 5% milk in TBS/Tween 20 for 1 hr at room temperature. Membranes were then incubated with the following primary antibodies, androgen receptor (AR), Bax, PERK, CHOP, IRE1α, BiP and caspase-4 overnight at 4°C and then with the corresponding HRP-labeled secondary antibodies for 1 hr at room temperature. The blots were developed with Super Signal West Femto chemiluminesent substrate (Thermo Scientific) and visualized using the FlourChemE Imager. All blots were probed with β-actin antibody to confirm equal loading of proteins.

### RT-PCR

22Rv1 and LNCaP cells were treated with 40 µg/ml rosemary extract or DMSO for 24 hrs. Total RNA was purified from control and treated cells. One step RT-PCR was performed according to the manufacturer’s instruction. One microgram of total RNA was used as template for each reaction. House-keeping gene GAPDH was used as an internal control. Primer sequences were: GAPDH forward 5′-ACC ACA GTC CAT GCC ATC AC-3′, GAPDH reverse TCC ACC ACC CTG TTG CTG TA-3′, XBP-1-A forward 5′-TTA CGA GAG AAA ACT CAT GGC C-3′, XBP-1-A reverse 5′-GGG TCC AAG TTG TCC AGA ATG C-3′.

### Immunofluorescence staining

Cells were cultured in 4-chamber slides (BD Biosciences) and treated with 0 and 50 µg/ml rosemary extract for 24hrs. After treatment, cells were washed once in PBS, fixed in 4% PFA for 15 mins and permeabilized in 0.2% Triton X-100 for 30 mins on ice. After blocking with 5% BSA in PBS for 1 hr, cells were incubated with CHOP antibody for 2 hrs followed by 1 hr treatment with corresponding fluorescent labeled secondary antibody. The slides were mounted and counterstained using Vectashield mounting medium containing DAPI (Vector labs). Cell images were taken at 40X magnification using Nikon microscope.

### Transfection

13nM control and CHOP siRNA and 50nM control and BiP siRNA were transfected in 22Rv1 and LNCaP cells using the *Trans*IT-siQuest reagent for 24 hrs. After siRNA transfection, cells were treated for another 24 hrs with 0 and 50 µg/ml rosemary extract and lysates prepared thereafter.

### Athymic nude mice study

All animal experiments were performed in accordance with the guidelines approved by the Animal Care and Use Committee of the University of Illinois at Chicago. The protocol was approved by the animal care committee at the University of Illinois at Chicago (Protocol Number: ACC-11-019). All animals were maintained under pathogen free conditions on a 12-hr light/dark cycle and fed with food and water ad libitum. 5 week old, 16 athymic J: NU outbred mice (Jackson Labs) were subcutaneously injected with 22Rv1 cells in the right and left flanks at a density of 1×10^6^cells/100 µl of RPMI + matrigel media. Mice were then divided into two groups of 8 mice each and the control group animals received 100 µl olive oil daily by oral gavage. The other 8 mice in the treated group received rosemary extract (100 mg/kg) dissolved in olive oil orally for 22 days. Body weights and tumor volumes were measured two times a week as described before [15]. Animal tissues were collected at the end of the study and stored at –80°C. Tissue lysates were then prepared in 1X RIPA buffer (Cell Signaling Technology) and its protein content measured using BCA assay kit (Thermo Scientific).Lysates were subjected to immunoblot analysis and probed for AR, PSA and CHOP.

### Statistical Analysis

All statistical analysis was done using GraphPad QuickCals software. Statistical comparisons between control and treated groups were performed by Student’s *t* test. All statistical tests were two- sided and P<0.05 was considered statistically significant.

## Results

### Standardization of Rosemary Extract

**Figure 1:**
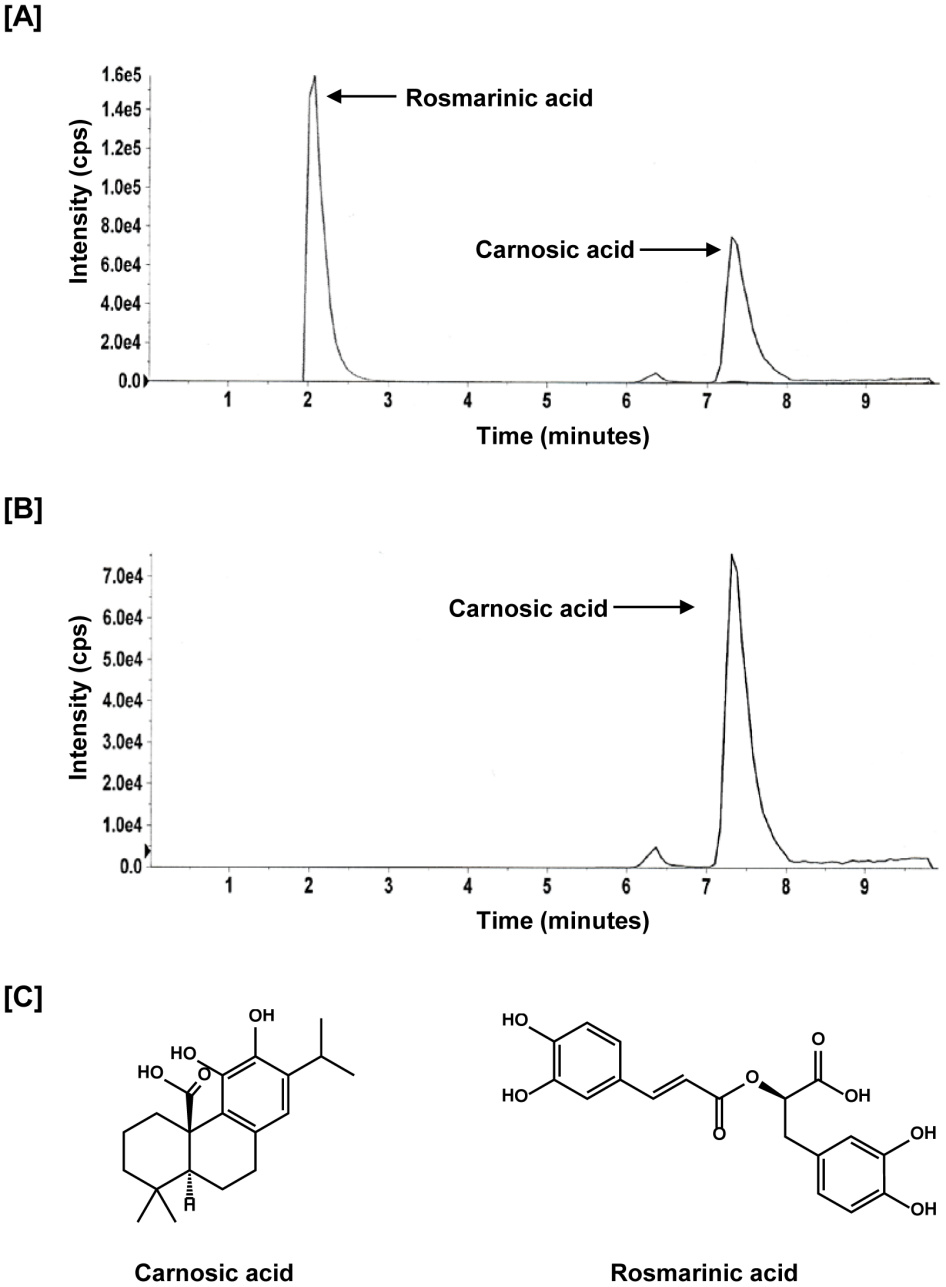
Standardization of Rosemary extract. A), Representative chromatogram of carnosic acid (analyte) and rosmarinic acid (internal standard) B), Representative chromatogram of carnosic acid at mass 331/287 C), Chemical structures of carnosic acid and rosmarinic acid.

For this study, we used an oil-soluble rosemary extract preparation containing carnosic acid and other phenolic diterpenes. Since carnosic acid accounts for majority of the antioxidant activity of rosemary extract we standardized the extract to carnosic acid using a LC/MS/MS method. The representative LC/MS chromatograms of carnosic acid and rosmarinic acid as internal standard are shown in Figure 1A. Figure 1B represents carnosic acid peak fragmented as a single ion at mass 331. LC/MS data showed 43% carnosic acid present in rosemary extract. The chemical structures of carnosic acid and rosmarinic acid are depicted in Figure 1C.

### Standardized Rosemary Extract decreases prostate cancer cell proliferation and viability

**Figure 2:**
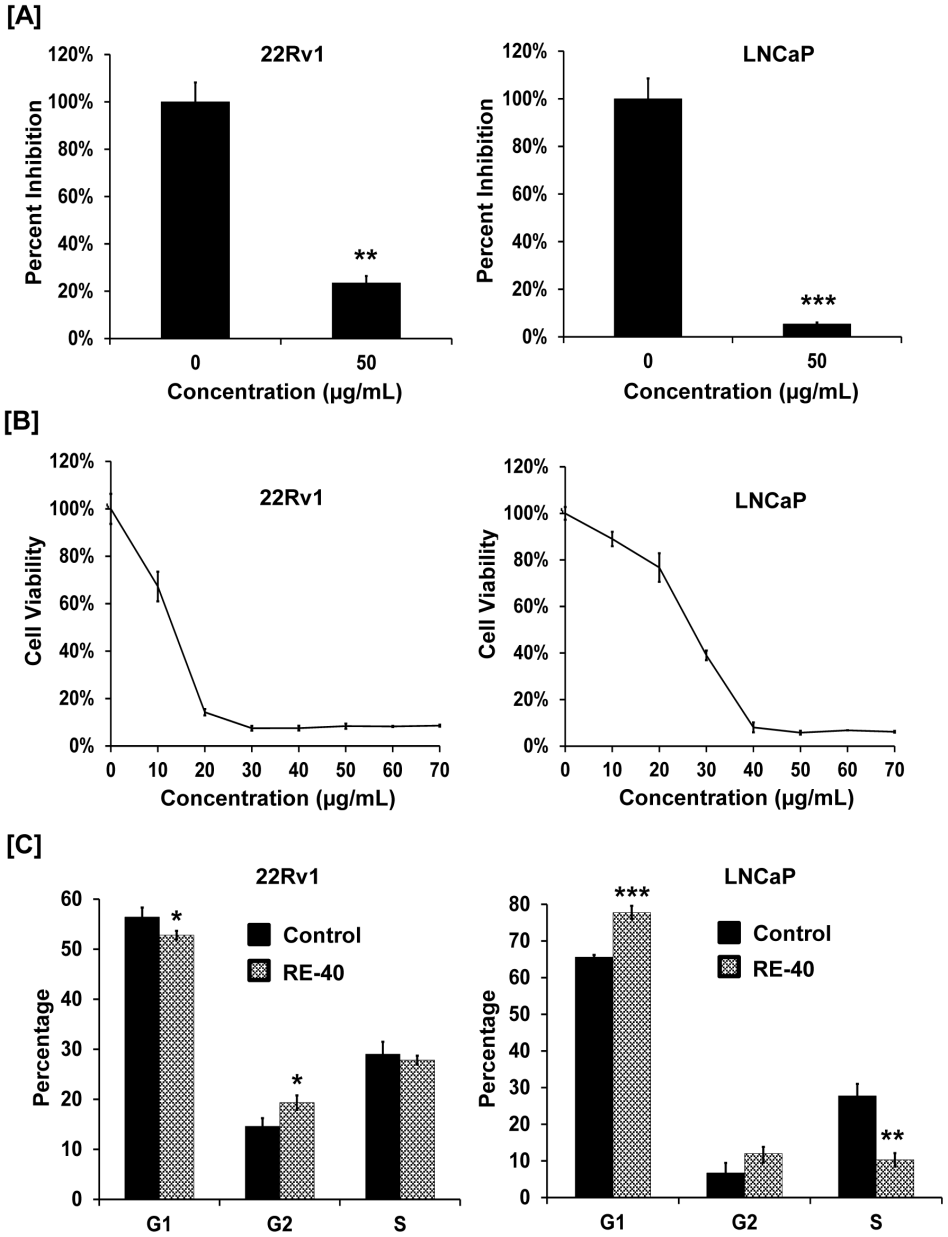
Rosemary extract modulates cell growth and induces cell cycle arrest in prostate cancer cell lines. A) 22Rv1 and LNCaP cells were treated with 0 and 50 µg/ml of Rosemary Extract standardized to 40% carnosic acid (RE-40) for 24 hrs. Cell proliferation was assessed by BrdU assay. B) Cell viability of 22Rv1 and LNCaP cells was determined using MTT assay. Cells were treated with increasing concentrations of RE-40 (0–70 µM) for 48 hrs. C) Effect of RE-40 treatment on cell cycle arrest in 22Rv1 and LNCaP cells. Cells were treated with RE-40 at 50 µg/ml for 24 hrs followed by labeling using the APODIRECT kit for flow cytometric analysis. Columns, results are representative of experiments performed in minimum of three replicates; bars, SD. *P<0.05, **P<0.01, ***P<0.001, RE-40 treated samples versus controls.

To determine the antiproliferative activity of rosemary extract we treated prostate cancer cells, 22Rv1 and LNCaP, with 0 and 50 µg/ml of rosemary extract for 24 hrs. As shown in Figure 2A, rosemary extract decreased proliferation of both 22Rv1 and LNCaP cells by 76.5 and 94.6% respectively. Likewise, we also observed a dose-dependent decrease in cell viability of 22Rv1 and LNCaP cells upon treatment with increasing concentrations of rosemary extract after 48hrs (Figure 2B). The IC_50_ values observed for 22Rv1 and LNCaP cells are 13.3 µg/ml and 27 µg/ml respectively.

### Standardized Rosemary Extract induces cell cycle arrest in prostate cancer cells

Treatment of 22Rv1 cells with 40 µg/ml rosemary extract for 24 hrs resulted in accumulation of cells in the G2 phase of the cell cycle whereas treatment of LNCaP cells resulted in both G1 and G2 cell cycle arrest as shown in Figure 2C.

### Standardized Rosemary Extract induces apoptosis in prostate cancer cells

**Figure 3:**
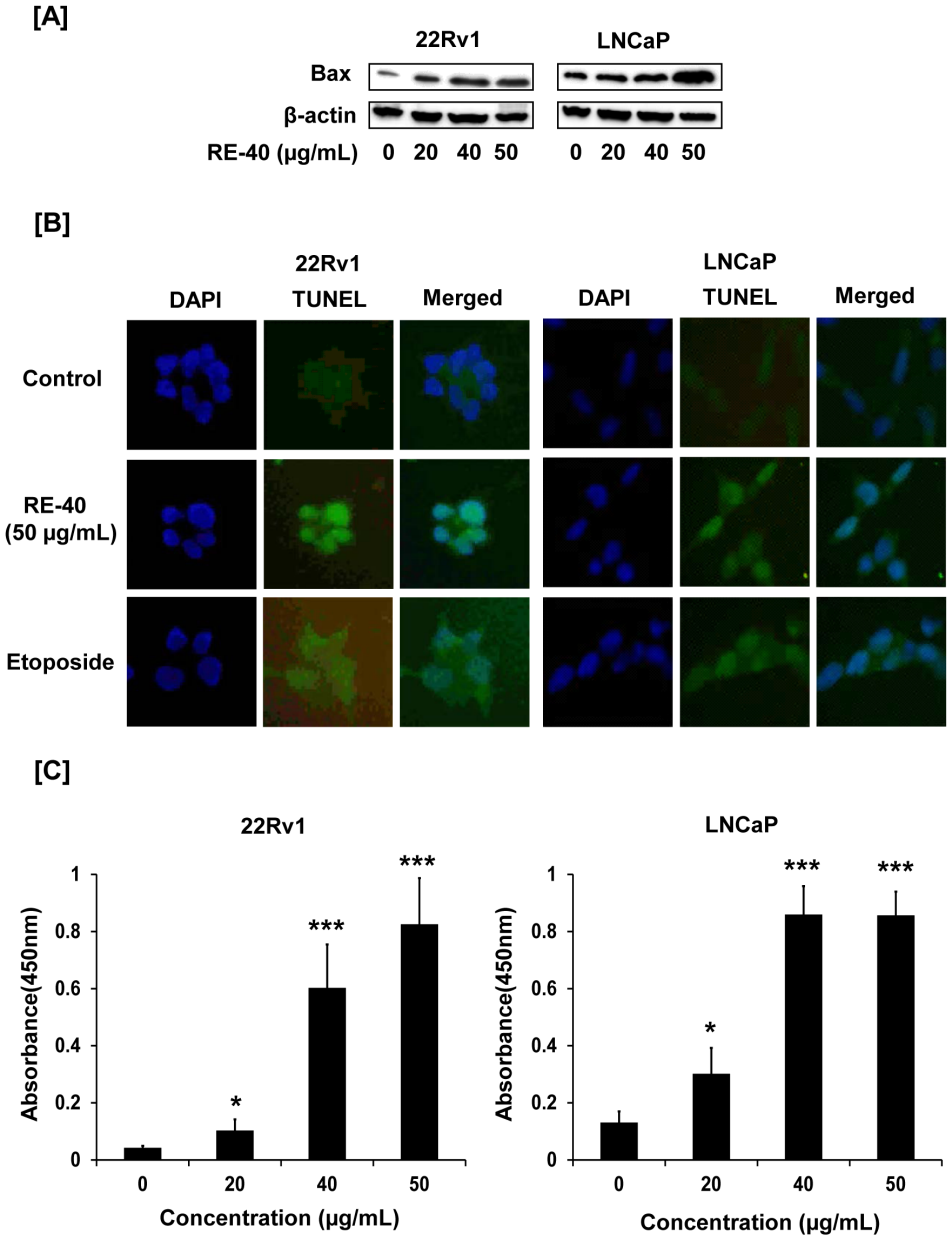
Rosemary extract promotes apoptosis in prostate cancer cells. A) 22Rv1 and LNCaP cells were treated with RE-40 for 24 hrs after which whole cell lysates were prepared and analyzed for expression of pro apoptotic protein, Bax, by Western blotting. Equal loading of protein was confirmed by probing with β-actin antibody. B) Apoptosis induction after 24 hrs of 0 and 50 µg/ml RE-40 treatments in 22Rv1 and LNCaP cells was assessed by TUNEL assay. For both cell lines the first column shows: DAPI-stained nuclei appear blue, second column: FITC-stained nuclei appear green showing induction of apoptosis, third column: merged picture of DAPI and FITC. Cells were also treated with etoposide as a positive control for apoptosis. C) Cleaved caspase-3 levels were detected by ELISA in 22Rv1 and LNCaP cells after treatment with 0, 20, 40 and 50 µg/ml RE-40 for 24hrs. Columns, results are mean of two different experiments performed in duplicate; bars, SD. *P<0.05, ***P<0.001, RE-40 treated samples versus controls.

To examine if rosemary extract promotes apoptosis we looked at the expression levels of pro-apoptotic protein, Bax, by Western blotting. With increasing concentrations of rosemary extract we saw an increase in expression of Bax in 22Rv1 and LNCaP cells (Figure 3A). To further confirm if rosemary extract induces apoptosis we performed a TUNEL assay on 22Rv1 and LNCaP cells. As shown in Figure 3B, 22Rv1 and LNCaP cells displayed green fluorescence for TUNEL staining upon treatment with rosemary extract at 50 µg/ml for 24 hrs compared to untreated control cells. Etoposide was used as a positive control for visualizing apoptosis. Additionally, we also detected increased production of cleaved caspase-3 in rosemary extract treated cells compared to untreated controls (Figure 3C). We observed a 20 fold and 6.6 fold increase in cleaved caspase 3 production in 50 µg/ml rosemary extract treated 22Rv1 and LNCaP cells respectively, compared to untreated control cells.

### Standardized Rosemary Extract decreases AR expression in prostate cancer cell lines

**Figure 4:**
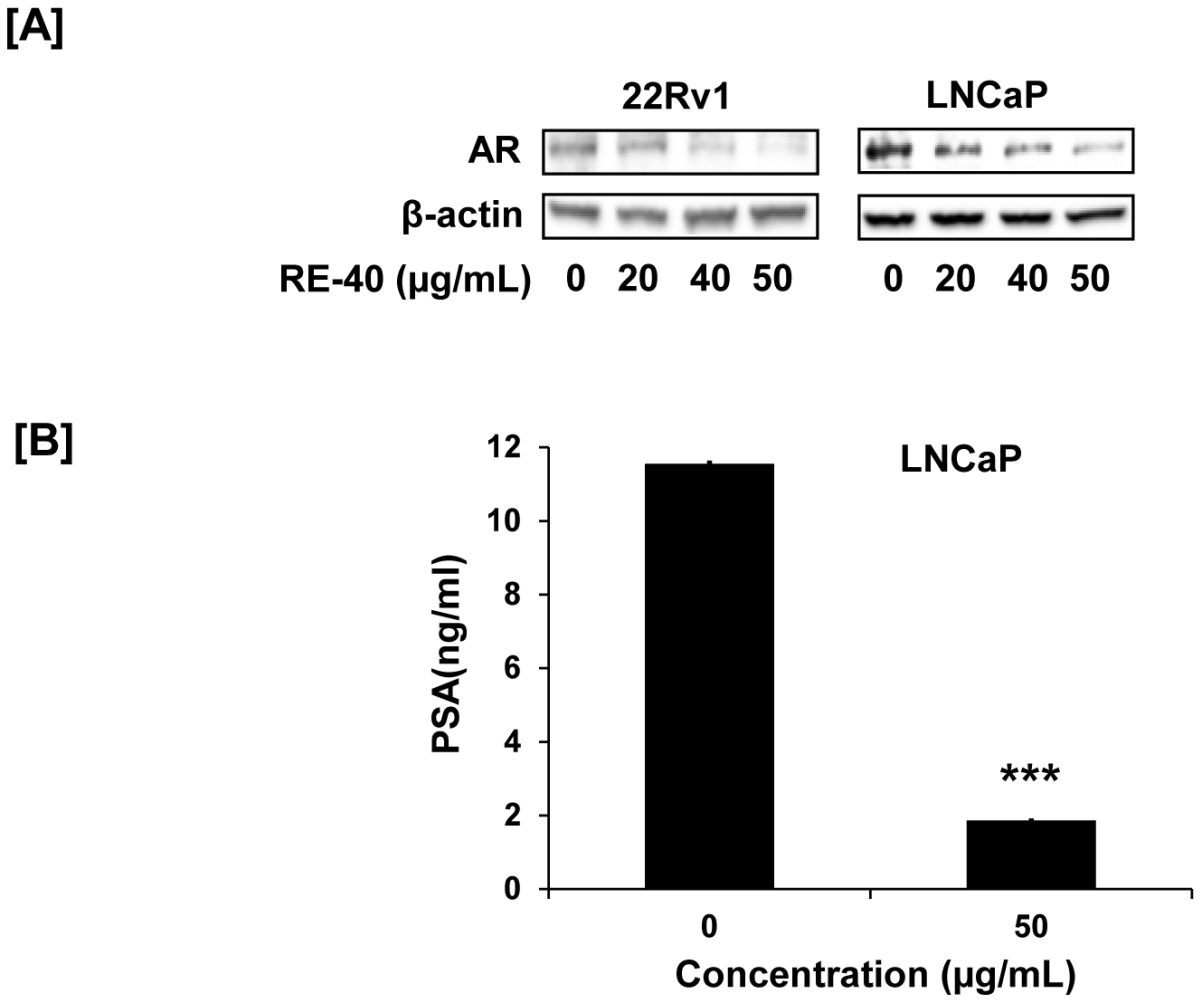
Modulation of AR and PSA proteins with Rosemary extract. A) 22Rv1 and LNCaP cells were treated with Rosemary Extract standardized to 40% carnosic acid (RE-40) for 24 hrs and cell lysates used to evaluate protein expression of AR by Western blot. B) Effect of RE-40 on PSA production in LNCaP cells after 24hrs was detected by ELISA. Columns, results are representative of experiments performed in six replicates; bars, SD. ***P<0.001, RE-40 treated sample versus control.

The androgen receptor has long been a target for prostate cancer. Hence we evaluated the expression of AR following treatment with rosemary extract in 22Rv1 and LNCaP cells. We observed a dose dependent decrease in expression of AR (Figure 4A) in prostate cancer cells with concentrations as low as 40 µg/ml and 20 µg/ml in 22Rv1 cells and LNCaP cells, respectively. Along with decreased AR expression we also observed 6 fold decrease in PSA protein secreted in culture media of LNCaP cells after treatment with 50 µg/ml of rosemary extract for 24 hrs as measured by PSA ELISA (Figure 4B)

### Standardized Rosemary Extract promotes endoplasmic reticulum (ER) stress in prostate cancer cells

**Figure 5:**
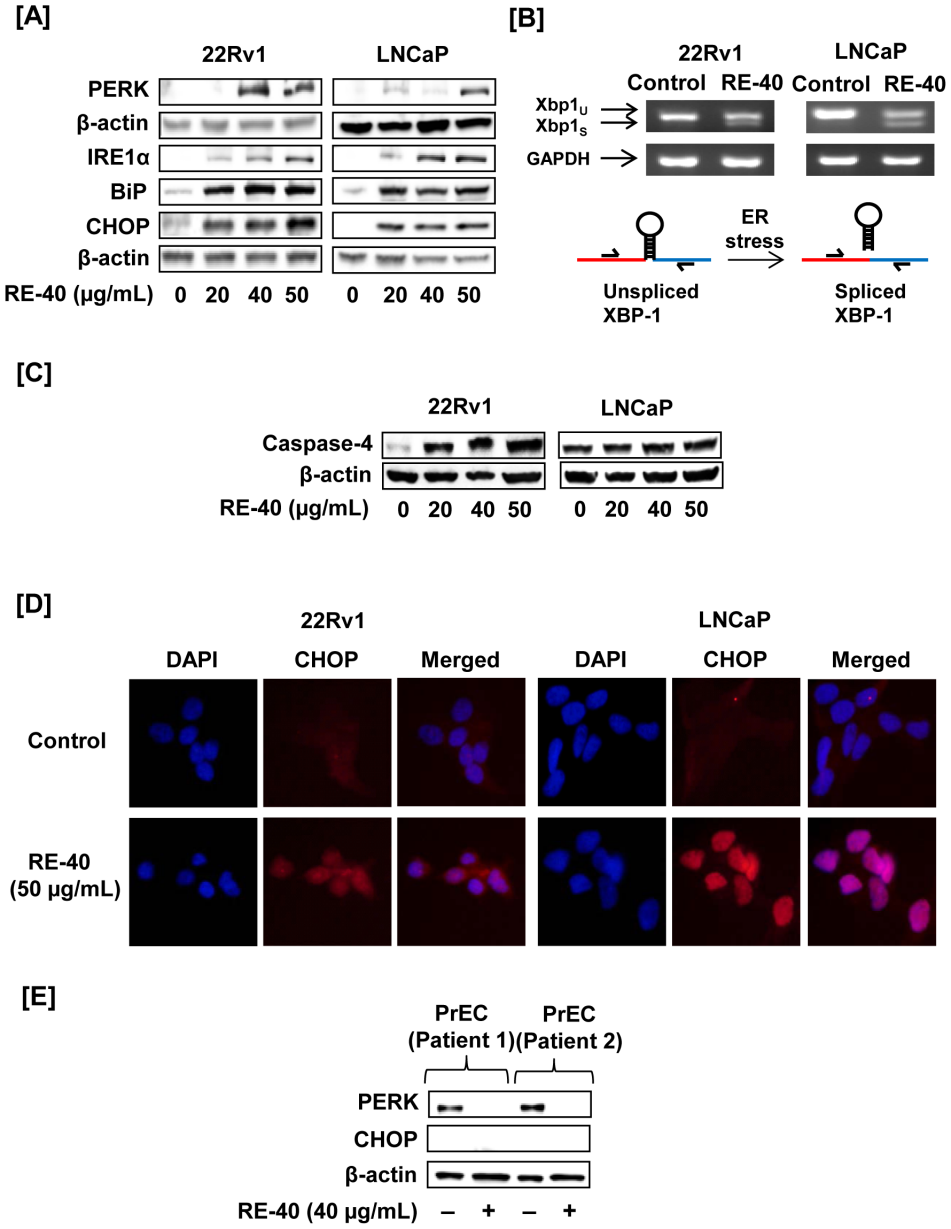
Rosemary extract modulates ER stress proteins. A) Cell lysates of 22Rv1 and LNCaP cells were prepared after treatment with RE-40 for 24hrs. Cell lysates were used to evaluate expression of ER stress proteins PERK, IRE1α, BiP and CHOP using Western blotting. Equal loading of protein was confirmed by probing the blot with β-actin. B) Splicing of XBP1 mRNA as an indicator of ER stress activation in both 22Rv1 and LNCaP treated cells. GAPDH was used as an internal control for RT-PCR. C) 22Rv1 and LNCaP cell lysates were tested for caspase-4 activation during ER stress by western blotting. D) Immunofluorescence staining for CHOP expression in 22Rv1 and LNCaP prostate cancer cells after treatment with 50 µg/ml of RE-40. For both cell lines the first column shows: DAPI-stained nuclei appear blue, second column: Tx-red stained nuclei appear red displaying expression of CHOP protein, third column: merged picture of DAPI and Tx-red. E) To determine the effect of RE-40 on human cells, prostate epithelial cells were treated with RE-40 and lysates confirmed for expression of ER stress protein, PERK and CHOP, by Western blotting

Next, we evaluated the expression levels of ER stress proteins PERK, IRE1α, XBP-1, BiP, CHOP and caspase-4 upon treatment with 0–50 µg/ml of rosemary extract in both 22Rv1 and LNCaP cells. As shown in Figure 5A, treatment with rosemary extract, resulted in an increase in PERK, often the first ER stress protein to be modulated. An increase in protein expression of IRE1α was observed at 20 µM. A significant increase in BiP, an ER stress chaperone protein, and CHOP was also observed upon treatment with 20 µM of rosemary extract (Figure 5A). In response to ER stress, activated IRE1α cleaves XBP1 mRNA to remove a 26-nucleotide intron and generate a translational frameshift mutation. Thus, using RT-PCR we tested for XBP1 splicing using primers specific for the 26-nucleotide intron. As shown in Figure 5B, rosemary extract treatment induced splicing of XBP-1 mRNA in both prostate cancer cell lines compared to untreated controls. GAPDH was used as an internal control. All upstream signals of UPR ultimately lead to activation of caspase-4 during ER stress. Our results show an increase in caspase-4 expression in both 22Rv1 and LNCaP cells upon treatment with rosemary extract (Figure 5C).

### Rosemary Extract modulates CHOP/GADD153

Next, we evaluated the ability of rosemary extract to modulate the ER stress protein CHOP/GADD153 *in vitro.* 22Rv1 and LNCaP cells were grown in chamber slides for 24 hours and treated with rosemary extract for 24 hours and prepared as described in Materials and Methods. We observed an increase in overall protein expression of CHOP (Figure 5D). This can be seen by the increased red fluorescence in CHOP panels along with the merged panel containing the counterstain with 4′,6-diamidino-2-phenylindole (DAPI). Being that CHOP is a transcription factor localized to the nucleus is consistent with its role in the unfolded protein response pathway. Observations were consistent in both 22Rv1 and LNCaP cells.

### Rosemary Extract selectively induces ER stress in prostate cancer cells while sparing normal prostate cells

Our next objective was to determine the potential of rosemary extract to promote ER stress in non-tumorigenic cancer cells. Prostate epithelial cells were isolated from benign peripheral zone in patients undergoing radical prostatectomy. Interestingly, we observed rosemary extract was unable to increase the expression of the critical ER stress proteins, PERK, in benign human prostate epithelial cells (Figure 5E). Interestingly, a decrease in PERK expression in prostate epithelial cells was observed following treatment with rosemary extract.

### AR degradation by Rosemary Extract is dependent on BiP

**Figure 6:**
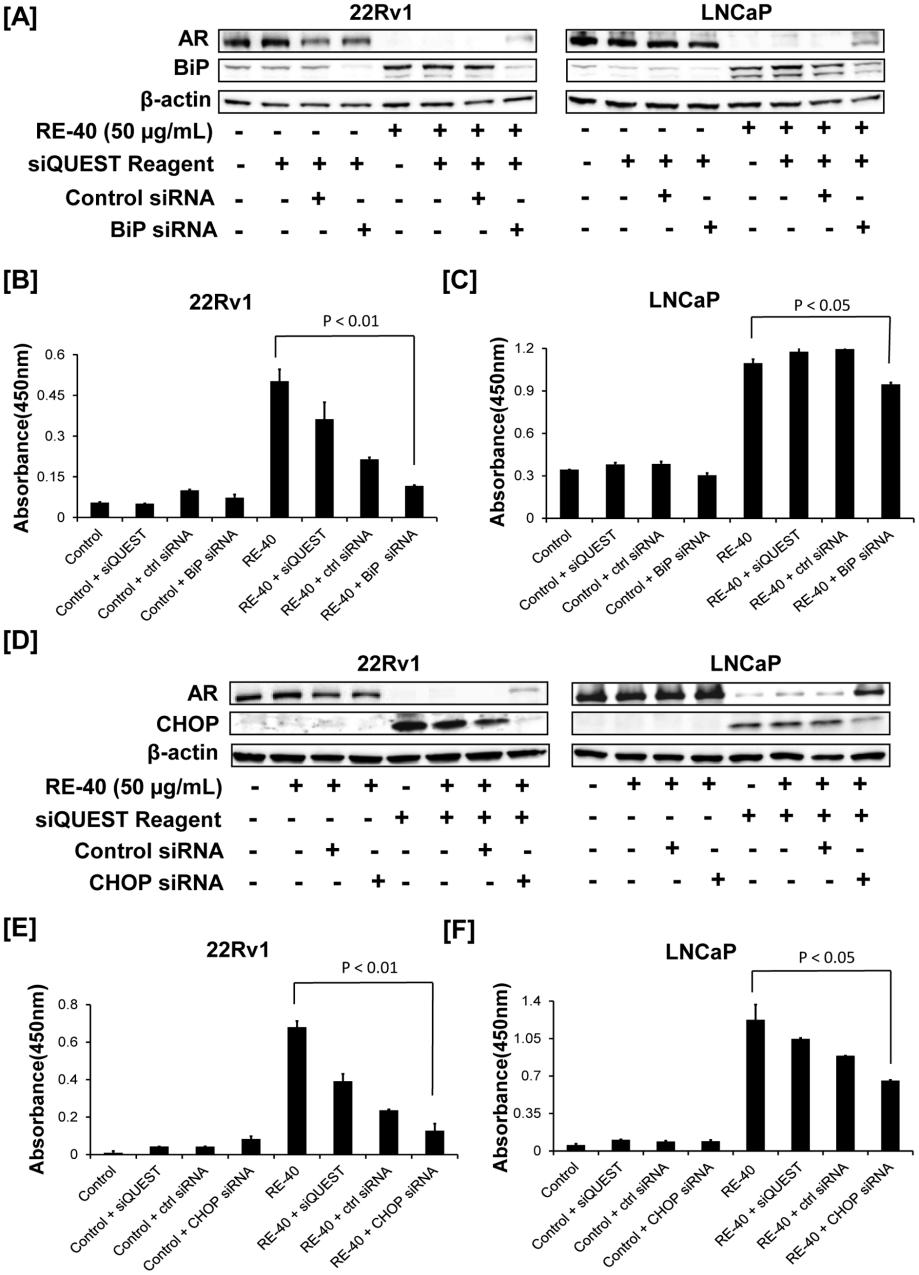
Rosemary extract modulates BiP and CHOP resulting in decreased AR expression. A). BiP siRNA treated cell lysates were prepared and checked for AR and BiP protein expression using Western blotting. Equal loading of protein was confirmed by probing the blot with β-actin. B) 22Rv1 cell lysates were used to detect protein levels of cleaved caspase 3 protein by ELISA as a marker for apoptosis. C) LNCaP cell lysates were used to detect protein levels of cleaved caspase 3 by ELISA as a marker for apoptosis. D) 22Rv1 and LNCaP cell lysates transfected with CHOP siRNA were prepared and checked for AR and CHOP protein expression using Western blotting. Equal loading of protein was confirmed by probing the blot with β-actin. E) 22Rv1 cell lysates were used to detect protein levels of cleaved caspase 3 protein by ELISA as a marker for apoptosis. F) LNCaP cell lysates were used to detect protein levels of cleaved caspase 3 by ELISA as a marker for apoptosis. Columns, results are representative of experiments performed in duplicate; bars, SD. Each experiment was repeated two independent times.

To understand the role of the ER stress chaperone protein BiP in AR degradation we treated 22Rv1 and LNCaP cells with rosemary extract with or without BiP-siRNA. As seen in Figure 6A, expression of BiP protein is increased in rosemary treated cells along with a decrease in AR expression. However, when cells were treated with a combination of BiP-siRNA and rosemary extract, androgen receptor degradation was reversed. These results suggest that BiP may have a critical role in regulating AR degradation (Figure 6A).

### BiP is a critical regulator in caspase induced apoptosis in Rosemary Extract treated prostate cancer cells

Next, we evaluated the role of BiP in regulating caspase-3 activation in 22Rv1 and LNCaP cells. As expected rosemary extract treatment induced activation of caspase-3 in both 22Rv1 and LNCaP cells. However, when cells were treated with combination of BiP siRNA and rosemary extract the activation of caspase-3 was decreased in both 22Rv1 and LNCaP cells (Figure. 6B and 6C respectively). These results suggest that rosemary extract induced BiP expression is essential for apoptosis.

### AR degradation by Rosemary Extract is dependent on CHOP/GADD153

22Rv1 and LNCaP cells were further evaluated to understand if CHOP is a key regulator in AR degradation. 22Rv1 and LNCaP cells were treated with rosemary extract with or without CHOP-siRNA. As expected a significant increase in CHOP expression and decrease in AR expression was observed following treatment with rosemary extract. However, when cells were treated with a combination of CHOP-siRNA and rosemary extract androgen receptor degradation was reversed. These results suggest that CHOP may have a critical role in regulating AR degradation (Figure 6D).

### CHOP is a critical regulator in caspase induced apoptosis in Rosemary Extract treated prostate cancer cells

Next, we evaluated the role of CHOP in regulating caspase-3 activation in 22Rv1 and LNCaP cells. We observed rosemary extract to increase activation of caspase-3 as observed earlier. Interestingly, when cells were treated with CHOP siRNA and rosemary extract the activation of caspase-3 was decreased in 22Rv1 cells (Figure 6E) and LNCaP cells (Figure 6F). These results suggest that rosemary extract induced CHOP expression is essential for apoptosis.

### Effect of Rosemary Extract on progression of prostate cancer *in vivo*

**Figure 7:**
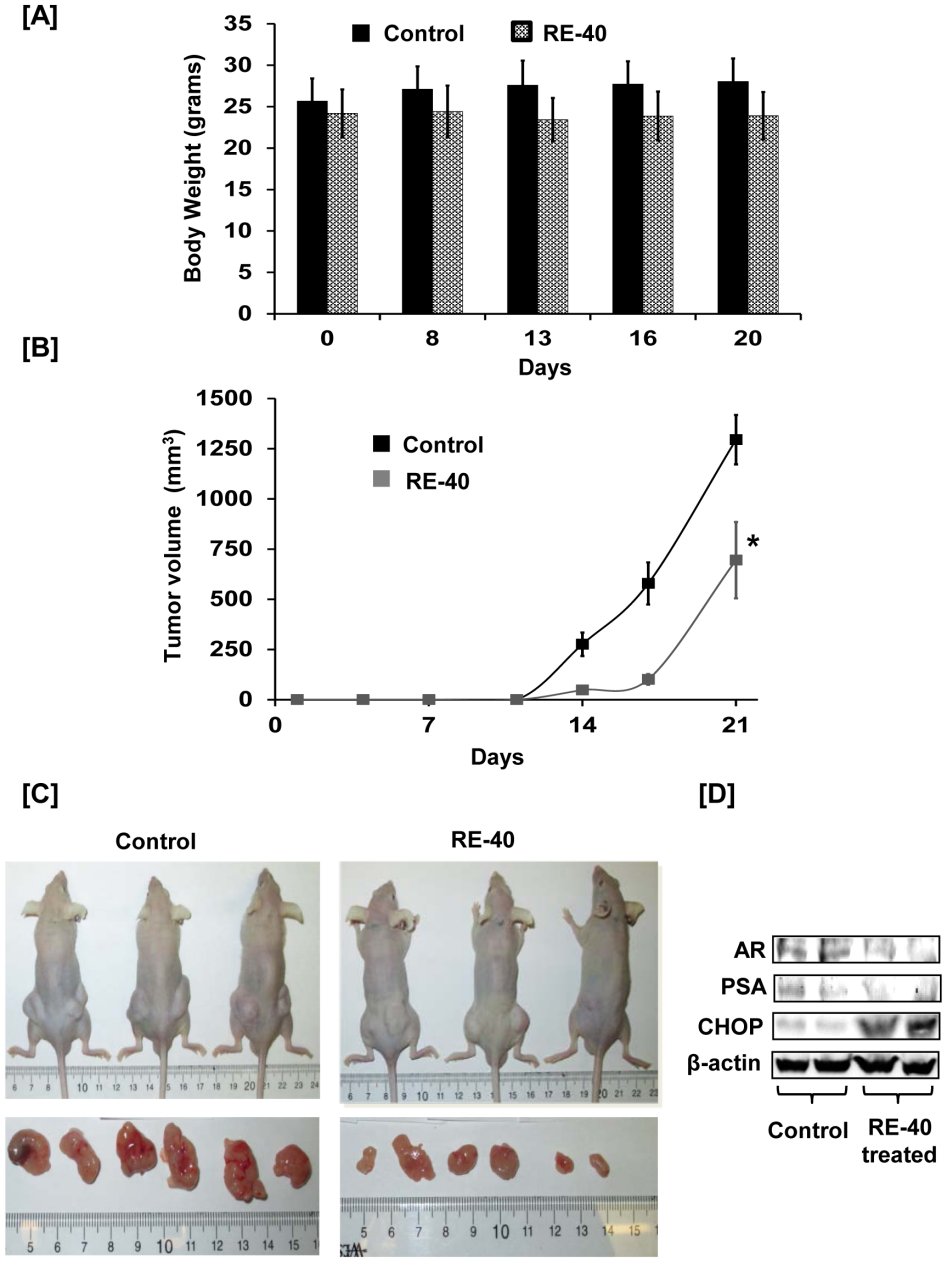
Rosemary extract decreases prostate cancer xenograft tumor growth in athymic nude mice. A) Graph of body weight measurements in grams for control and RE-40 treated animals against number of days of 22Rv1 tumor inoculation. B) Average tumor volume (mm^3^) of both control and RE-40 treated mice plotted over days of tumor cell inoculation. C) Representative pictures of control and RE-40 treated mice and tumors removed from them. D) Mouse tissue lysates were prepared from control and rosemary extract treated groups and analyzed for expression of AR, PSA, CHOP and β-actin by western blotting.

Athymic nude mice were administered either olive oil alone or rosemary extract dissolved in olive oil to determine the efficacy of rosemary extract on progression of prostate cancer *in vivo*. As evident from their body weight measurements, mice tolerated rosemary extract well for 22 days (Figure 7A). Development of tumors was observed at day 14 in both groups of mice, with a smaller volume observed in mice treated with rosemary extract as shown in Figure. 7B. At day 21, tumors in the treated group measured 695 mm^3^ whereas tumors from control mice measured 1295 mm^3^ depicting a 46% reduction in tumor size in rosemary extract treated mice compared to control animals (Figure. 7B). A representative image of control and rosemary extract treated mice along with pictures showing different dimension of tumors from each group is shown in Figure 7C. We also analyzed mouse tissue lysates using Western blotting for expression of AR, PSA and CHOP. As shown in Figure 7D, expression of AR and PSA is decreased and that of CHOP is increased in rosemary extract treated tissue lysates compared to lysates from control group animals.

## Discussion

The Mediterranean diet has been receiving attention for its cardiovascular and metabolic health promoting properties [16], [17]. In particular, fresh fruits and vegetables, legumes, non-refined cereals, fish, olive oil, and red wine have received the most attention for their health promoting properties [18]. One area that has not received enough attention is the use of culinary herbs including rosemary, basil, oregano, sage, and others [19]. Specifically, rosemary (*Rosmarinus officinalis*) has been associated with anti-inflammatory and anti-oxidant properties. Previously, we have reported carnosol, an ortho diphenolic diterpene, to promote apoptosis in multiple prostate cancer cell lines while modulating 5' adenosine monophosphate-activated protein kinase (AMPK) pathway [20]. More recently, we have reported a unique attribute of carnosol to promote dual disruption of AR and ERα [15].

In this particular study, we evaluated rosemary extract standardized to carnosic acid for decreasing prostate cancer cell viability. The rationale for evaluating a complex mixture of rosemary polyphenols was several fold [19]. First, several epidemiological studies have reported that rosemary consumption is associated with an overall reduction in cancer incidence [21]. Second, the European Union has approved the use of rosemary extracts for food preservation and has been adopted into the EU food legislation [22]. Third, in the United States rosemary has been categorized by the FDA as “generally recognized as safe” or GRAS (CFR 182.10; 182.20). Fourth, rosemary is widely available as a dietary supplement in the United States. Lastly, it is apparent from our studies as well as others that complex mixtures of polyphenols under certain circumstances can provide a multi-targeted effect compared to individual phytochemicals. Based on these observations as well as our previous evaluation of individual phytochemicals led us to investigate rosemary extract standardized to carnosic acid.

Alterations in the environment surrounding the endoplasmic reticulum have a direct effect on the integrity, structure and function of this organelle [7], [8]. This is especially significant during cancer promotion and progression as there is an increased demand on the protein production of the endoplasmic reticulum to meet the increased metabolic needs of cancer cells. During this process it is evident that there is an increased likelihood of proteins that are misfolded or even unfolded. If left unchecked, these cells will undergo apoptosis. In response, the cell has the capability to address protein instability via the “unfolded protein response” which is controlled by PERK and CHOP, along with several other ER stress proteins [23]. As the cancer cell compensates for this established homeostasis, proteins may fold properly or undergo a controlled degradation thereby increasing cell survival. Evidence by other investigators and ourselves suggests that the use of small molecules to modulate ER stress may delay tumor development, growth, invasion, thereby providing a novel therapeutic strategy [24]. As reported above we observed rosemary extract to modulate ER stress proteins, including CHOP/GADD153. The effects of modulating CHOP/GADD153 can be seen as early as 20 µg/ml of rosemary extract (40% carnosic acid) which approximates to 24 µM of carnosic acid. Based on previous reports of the pharmacokinetic parameters of carnosic acid it would appear that these concentrations are achievable following oral administration [25], however, further work would be required to determine the pharmacokinetic profile in mice [26].

On another front in prostate cancer control it is well known that the androgen receptor has been a target for prostate cancer for a long time. It is becoming increasingly clear that there is a significant shortcoming with targeting the androgen receptor with traditional ligand binding receptor antagonists. First, the role of anti-androgens is often limited because androgen antagonists (e.g. flutamide and bicalutamide) are used to control the initial prostate cancer “flare” [27]. This is often followed by an eventual conversion of prostate cancer to a castration resistant form. The response of the cancer cell to “resist” anti-androgens is correlated with mutations in the androgen receptor that render traditional anti-androgen antagonists ineffective or even counter-productive.

The evidence is overwhelming that the androgen receptor is a valuable target for prostate cancer, however, an alternative strategy should be explored to disrupt the androgen receptor as opposed to traditional antagonists at the ligand binding domain. One approach that has been proposed is targeted degradation of the androgen receptor, however, at present these chemicals are too toxic for *in vivo* studies. In our study we observed rosemary extract to decrease androgen receptor protein expression at concentrations as low as 20 µg/mL (∼ 24 µM of carnosic acid). Furthermore we observed a significant decrease in secreted prostate specific antigen in LNCaP cells from 11.5 to 2 ng/ml with rosemary extract treatment. The next step was to determine if there was a link between CHOP/GADD153 and decreased androgen receptor with rosemary extract treatment. In both 22Rv1 and LNCaP cells we observed a significant increase in CHOP, however, using siRNA along with rosemary extract treatment we observed a reversal of rosemary extract induced androgen receptor degradation. However, given that CHOP is generally considered a downstream event in the ER stress response as it closely regulates the anti-apoptotic and pro-apoptotic proteins we wanted to evaluate proteins earlier in the ER stress response. Next, we performed siRNA with BiP, an ER stress chaperone, with or without rosemary extract. Interestingly, when cells were treated with BiP siRNA and rosemary extract a reversal of the AR degradation is observed. In regard, to the function of BiP it will go through multiple cycles of binding and releasing a substrate protein through an ATP driven process that will allow the substrate to fold properly [28]. Under those conditions protein disulfide isomerase (PDI) is recruited to reduce, rearrange, and oxidize disulfide bonds until the correct conformation is achieved. Interestingly, prolonged binding of BiP to a protein has been shown to result in proteasomal degradation [29]. Another interesting observation of BiP by others has shown it to be a key regulator of translocon pores in the endoplasmic reticulum [28]. Future work will need to evaluate if rosemary extract is responsible for governing the pore and/or ushering proteins to the proteasome.

Two benign primary PrE cell lines were evaluated to understand if a bisphasic response in ER stress modulation would be observed. In these cells significant upregulation of PERK was observed suggesting that these cells, although from benign regions of the prostate, are at a critical turning point in the carcinogenic process possible as a result of age or field effects [13]. These PrE cells also do not express androgen receptor, which may contribute to the differential response to rosemary extract, resulting in a decrease in PERK expression. This biphasic response further illustrates the complexity of ER stress during prostate carcinogenesis. It is clear that rosemary is an abundant source of a variety of different polyphenolic substances that are different than other well-known natural products and will require a more detailed understanding of how these chemical constituents can contribute to ER stress and androgen receptor degradation [20], [30].

The accumulation of mutations in “normal” cells results in an inability to overcome traditional checkpoints is critical in prostate carcinogenesis. Based on the results described above further work is needed to understand how the individual phytochemicals in rosemary modulate critical targets that regulate the cell cycle and endoplasmic reticulum stress. Especially within prostate cancer it is evident that the androgen receptor which acts as a transcription factor for over 800 genes is a critical component of this process. For this reason, the androgen receptor has been aggressively targeted through androgen receptor antagonists, however, a new approach may be needed to overcome the traditional obstacles that present themselves shortly after therapy initiation. In our studies, we observed rosemary extract to selectively induce ER stress proteins and provide evidence that CHOP/GADD153/BIP is a critical pathway of androgen receptor degradation. Furthermore, these results were also observed in 22Rv1 xenograft mouse tissues following oral administration of rosemary extract. Interestingly, in normal prostate epithelial cells procured from patients at high risk of developing prostate cancer rosemary extract standardized to carnosic acid is observed to decrease ER stress. Further work will be required to evaluate individual diterpenes in modulating ER stress proteins in prostate cancer to further understand how rosemary extract promotes ER stress.
